# Motor performance and functional ability in preschool- and early school-aged children with Juvenile Idiopathic Arthritis: a cross-sectional study

**DOI:** 10.1186/1546-0096-6-2

**Published:** 2008-01-16

**Authors:** Janjaap van der Net, Patrick van der Torre, Raoul HH Engelbert, Vivian Engelen, Femke van Zon, Tim Takken, Paul JM Helders

**Affiliations:** 1 Dept. of pediatric physical therapy and pediatric exercise physiology, Children's Hospital at the University Medical Centre at Utrecht, Suite: KB 02.056.0, PO Box 85090, 3508 AB Utrecht, The Netherlands

## Abstract

**Objective:**

To describe the level of motor performance and functional skills in young children with JIA.

**Methods:**

In a cross-sectional study in 56 preschool-aged (PSA) and early school- aged children (ESA) with JIA according to ILAR classification, motor performance was measured with the Bayley Scales of Infant Development II (BSID_2_) and the Movement Assessment Battery for Children (M-ABC). Functional skills were measured with the Pediatric Evaluation of Disability Inventory (PEDI). Disease outcome was measured with a joint count on swelling/range of joint motion, functional ability and joint pain.

**Results:**

Twenty two PSA children (mean age 2.1 years) with a mean Developmental Index of the BSID_2 _of 77.9 indicating a delayed motor performance; 45% of PSA children showed a severe delayed motor performance. Mean PEDI scores were normal, 38% of PSA scored below -2 SD in one or more domains of the PEDI. Thirty four ESA children (mean age 5.2 years) with a mean M-ABC 42.7, indicating a normal motor performance, 12% of ESA children had an abnormal score. Mean PEDI scores showed impaired mobility skills, 70% of ESA children scored below -2 SD in one or more domains of the PEDI. Disease outcome in both age groups demonstrated low to moderate scores. Significant correlations were found between age at disease onset, disease duration and BSID_2 _or M-ABC and between disease outcome and PEDI in both age cohorts.

**Conclusion:**

More PSA children have more impaired motor performance than impaired functional skills, while ESA children have more impairment in functional skills. Disease onset and disease duration are correlated with motor performance in both groups. Impaired motor performance and delayed functional skills is primarily found in children with a polyarticular disease course. Clinical follow up and rehabilitation programs should also focus on motor performance and functional skills development in young children with JIA.

## Background

Juvenile Idiopathic Arthritis (JIA) is the most common chronic joint disease in childhood [1]. The disease can lead to physical disability and reduced health-related quality of life [2]. Retrospective long-term follow-up studies have revealed significant levels of disability in adolescence and adulthood JIA [3-5]. For this reason functional status in JIA became an important outcome in clinical trials and health care programs [6]. There is little knowledge so far about the development and mastering of functional activities in young children with JIA. Miller et al. reported in a retrospective study of 88 school age children with Juvenile Rheumatoid Arthritis (JRA; now referred to as JIA) that children with JRA have significantly impaired physical function compared to healthy children, including those with pauci-articular JRA (now referred to as oligo-articular JIA) [7]. This seems relevant as school-age children are expected to have mastered a set of motor- and functional skills that allow them to fully participate in school activities, play and physical education. From this perspective it is of interest whether young children with JIA have a delayed motor performance and/or a delay in acquiring functional skills development and whether this is related to age at disease onset, disease course and disease severity. Only one case report by Hollister describes the changes in motor behaviour of two children with JIA, and concludes that the motor milestones in these children were achieved at the same time as in their healthy peers [8]. However, currently there are no studies that describe the development of motor performance and functional skills and its relationship with age at disease onset, disease course and disease severity in young children with JIA.

Therefore the purpose of this study was to describe the level of motor performance and the level of functional ability in relationship to the age of disease onset, disease course and disease outcome, in a group of preschool- and early school- aged children with JIA.

## Methods

Fifty-six consecutive children under the age of 7 years, regularly visiting the outpatient clinic of a tertiary center for pediatric rheumatology were included in this study. All children were classified according to the ILAR criteria [9]. All parents gave informed consent. Children with multiple diagnoses were excluded from this study.

Disease history was taken retrospectively from the medical charts. Disease outcome parameters for this study were joint swelling and joint range of motion, functional ability and reported measures of discomfort. To allow for easy comparison all disease outcome parameters are presented as percentage of maximal possible scores. Joint swelling was scored for the sterno-clavicular-, glenohumoral-, elbow-, wrist-, hand-, hip-, knee-, ankle- and foot joints bilaterally (hand and foot joints included one or more metacarpal or metatarsal and phalangeal joints). Classification for joint swelling was performed on a four point Likert scale (score range 0–3; total scale 0–54) [10]. Range of joint motion was scored on the Pediatric Escola Paulista de Medicina Range of Motion Scale (pEPM-ROM) using a two-legged goniometer [11]. The pEPMROM measures 10 motions (flexion/extension, abduction or rotation) in 8 joints bilaterally (cervical spine, shoulder, wrist, hand, hip, knee and ankle). Joint limitation for each motion was classified on a 4-point Likert scale ranging from no – 30% limitation in ROM (score range 0–3, total scale 0–60). Joint status was assessed by experienced pediatric physical therapists (RHHE, PvdT, JvdN) during a routine outpatient clinic visit at the department of pediatric physical therapy. Functional ability was assessed with the Dutch version of the Childhood Health Assessment Questionnaire parent form (CHAQ) [12,13]. The CHAQ consists of eight domains with 2 or more items representing common childhood daily activities. Each domain is represented by its worst item-score. The mean of the sum of scores on the eight domains represents the CHAQ-disability score (range of the scale 0–3). Perceived joint pain and perceived disease severity are scored with a double anchored Visual Analogue Scale and this represents the CHAQ-discomfort score. To study the motor performance two different, reference based, outcome measures had to be used as there are no instruments that include an age span from 1–7 years. We therefore stratified the patient group in two cohorts; the preschool age children (PSA) from 12–42 months of age and the early school age children (ESA) from 4.0–6.9 years of age. In PSA children the motor scale of the Dutch translation of the Bayley Scales of Infant Development II (BSID_2_) was used [14,15]. This test consists of a structured observation scale of a variable number of motor behaviours, such as locomotor (e.g. walking, stair climbing) and (bi) manual activities (e.g. drawing, stacking cubes), progressive in difficulty depending on age. From the item-sumscore a Developmental Index (DI) was calculated using original references from a population of children of the United States of America [14]. Normal DI's are defined as the range of +/- 2 standard deviations (score range: 70 – 130) [14]. A DI between -1 and -2 SD is considered a delayed motor development, a DI below -2 SD's is considered a severe delay in motor performance.

Motor performance in ESA children was assessed using the Dutch translation of the Movement Assessment Battery for Children (M-ABC) [16]. This test consists of 8-items measuring manual dexterity (e.g. drawing figures, lacing beads), ball skills (e.g. catching and bouncing) and dynamic and static balance (e.g. 1-leg standing, jumping a rope). In these categories, item scores can be calculated into sum scores which can be transformed into a percentile score based on a normative sample of an USA population [16]. A percentile score above the fifteenth percentile (> P15) has been defined as 'normal motor development'. A percentile score between the fifth (P5) and the fifteenth percentile (P15) has been defined as 'at risk for motor delay'. A percentile score below the fifth percentile (< P5) has been defined as a 'severe delay in motor development'. Functional skills was scored using the Dutch version of the Pediatric Evaluation of Disability Inventory (PEDI) [17]. The PEDI is a norm-referenced outcome measure for functional ability based on a pencil and paper questionnaire (197 items) for parents and/or caregivers. The PEDI has shown to be a reliable and valid generic instrument in a JIA population as well [18]. The instrument consists of two major independent dimensions (functional skills scales and caregiver assistance scales). For this study the functional skills scales have been used consisting of three content domains: self-care, mobility, and social function. The mastering of functional tasks and activities from the above mentioned domains is measured by this scale and is expressed in standard scores. Reference values are provided from a population from the United States of America for children between 0.5 and 7.5 years. Normal values are defined in the range of +/-2 standard deviations (normal range: 30 – 70). Data for the motor performance and functional skills are presented as mean (SD; range). Correlations were calculated (Spearman's Rho) between disease course, disease outcome and motor-/functional skills. All data were entered into SPSS data entry 3.0 and analyzed using SPSS-base 10.0 for Windows (SPSS Inc, Chicago, IL, USA).

## Results

A total of 56 children (36 girls and 20 boys) between 1.1 – 6.8 years of age were included in this study. At the time of this study all children were treated in a tertiary setting for pediatric rheumatology. In the PSA group, 22 children (14 girls and 8 boys) were studied with a mean age of 2.1 years (SD 0.5; range 1.1 – 3.1). The mean age of onset of disease was 1.7 years. (SD 0.5; range 1.0 – 3.0) and mean duration was 0,9 years. (SD 0.2; range 0 – 1.0). Six children had oligo-articular JIA, 15 had polyarticular JIA and one child had systemic onset JIA with a polyarticular disease course. All children were on anti inflammatory or immuno suppressive medication. Disease duration, disease outcome, motor development and development of functional skills in the disease subtypes in PSA children are presented in Table 1. Overall disease outcome (joint swelling-, pEPMROM- and CHAQ-scales) in PSA children demonstrated low to moderate scores (between 5% and 25%). The mean motor performance on the BSID_2 _(DI) in PSA children was 77.9 (SD 21.2; range 55–120), on group level indicating delayed motor development. Ten children (45%) had a severe delayed motor development as indicated by an abnormal DI on the BSID_2 _(< 70; i.e. < – 2 SD); all of these had a polyarticular clinical manifestation. Five children (23%) had a delayed motor development with a DI varying from 70 to 85, of which two had an oligo-articular disease and three a polyarticular clinical manifestation. One PEDI form was inadequately filled out and therefore not included, consequently data of 21 patients were included for analysis. The mean standard scores for the functional skills level on the PEDI domains selfcare, mobility and social skills are 44.1 (SD 6.3), 40.6(SD 14.1) and 46.5 (SD 13.8) respectively, indicating that the mean of functional skills in this patient group were all within normal limits. However, five children (23%) scored below -2 SD in one or more domains of the PEDI functional skills scale resulting in 6 scores < -2 SD and one child with oligo-articular JIA, four children with polyarticular JIA. On the PEDI self- care domain all children scored above -2 SD. On the PEDI mobility domain four children (18%)(one oligo- and 3 polyarticular) scored below -2 SD. On the PEDI-social skills domain two children (both polyarticular) scored below -2 SD. In ESA children, 34 children (22 girls and 12 boys) were studied with a mean age of 5.2 years (SD 0.9; range 4.0–6.8), of which 12 children had oligo-articular JIA, 14 children had polyarticular JIA, 6 children had systemic onset JIA and 2 children with extended oligo-articular JIA. The mean age of onset of disease was 3.0 years (SD 1.4; range 0.9–5.9) and mean duration was 5.6 years. (SD 1.6; range 0.1 – 5.6). The clinical characteristics of ESA children are presented in Table 2, 3. One child could not be tested on the M-ABC due to anxiety. There for 33 tests were included for analysis. The mean motor development on the M-ABC in ESA children was 42.7 (SD 32; range 1–96) in percentiles, indicating a normal motor performance in this patient cohort. However, four children (12%) had an abnormal score, i.e. a score below the 5^th ^percentile (one oligo-, one extended oligo- and two polyarticular JIA). Seven children (21%) (two oligo-, two systemic onset-, three polyarticular JIA) were considered at risk for an abnormal motor development, i.e. a score between 5^th ^percentile and 15^th ^percentile. Nine of these 11 cases had the poorest score on the Static & Dynamic Balance subscale, i.e. the gross motor function domain. One child scored poor on the Ball Skills subscale (speed & dexterity), and one had a poor score on the Fine Motor scale (i.e. eye-hand coordination & fine motor tasks). The mean functional skills level for the whole cohort of early school age children on the PEDI domains self-care, mobility and social skills were 40.5 (SD 8.2), 28.7(SD 14.7) and 43.5 (SD 11.7) respectively, indicating primarily an impairment of mobility skills. Twenty- one children (62%) scored below -2 SD in one or more domains of the PEDI functional skills, ten children with oligo-articular JIA, five children with polyarticular JIA, four children with systemic onset JIA and two children with extended oligo-articular JIA. On the PEDI self-care domain four children (12%) (one oligo-, two poly- and one systemic onset JIA) scored below -2 SD. On the PEDI mobility domain, 18 (55%) children (eight oligo-, five poly-, four systemic onset and one extended oligo-articular JIA) scored below -2 SD, reflecting that mobility was the most impaired of all functional skills. On the PEDI social skills domain, four children (one oligo-, two poly-, and one systemic onset JIA) scored below -2 SD.

**Table 1:** Disease outcome, level of motor performance and functional skills development in pre-school aged children with oligo-, polyarticular and systemic onset Juvenile Idiopathic Arthritis.

**Preschool age**													
OligoJIA n = 6					Score n < -2sd	PolyJIA n = 15					Score n < -2sd	SystJIA n = 1	Score n < -2sd

	Min	Max	Mean	SD			Min	Max	Mean	SD			

*Disease duration (yrs.)*	.0	1.0	.8	.4			1.0	1.0	1.0	.0		1.0	
*Disease Outcome (%):*													
Swelling index	0	55	13.3	20.9			0	40	18.5	13.4		10	
ped. EPM-ROM	0	10	5.7	4.1			2	25	11.1	5.7		10	
CHAQ Ds	0	35	13.3	13.3			0	75	27.3	22.3		15	
VAS pain	0	45	16.7	16.9			0	75	24.6	27.6		0	
VAS dis. severity	0	70	25	24.1			0	90	33.6	29.4		25	
													
*Motor skills:*													
BSID_2 _(DI)	79	120	99.8	16.7			55	108	70.33	16.9	9	60	1
													
*Functional skills*						n = 14							
PEDI Self-care	40.8	53.1	47.8	4.1			32.7	55.1	42.3	6.6		46.4	
PEDI Mobility	20.3	68.3	47.1	16.4	1		12.8	62.9	38.6	12.9	3	30	
PEDI Social skills	40.1	58.2	50	7.4			19.1	70.4	45.9	15.8	2	33.8	

OligoJIA: oligo-articular Juvenile Idiopathic Arthritis, PolyJIA: polyarticular Juvenile Idiopathic Arthritis, SystJIA: Systemic onset Juvenile Idiopathic Arthritis, ped. EPM-ROM: pediatric Escola Paulista Medicina Range Of Motion Scale, CHAQ Ds: Childhood Health Assessment Questionnaire Disability Score, VAS:

Visual Analogue Scale, Dis. Severity: Disease Severity. Disease outcome is presented in % of maximum loss/impairment. BSID2 (DI): Bayley Scale of Infant Development II (Developmental Index; presented as index scores, +/- 2 SD's is 70–130), PEDI: Pediatric Evaluation of Disability Inventory (presented as standard scores, range 30–70). Abn. Score: Abnormal Score (i.e. < -2 SD). n < -2 SD: number of patients with abnormal score.

**Table 2:** Disease outcome, level of motor performance and functional skills development in early school aged children with oligo-, polyarticular and systemic onset Juvenile Idiopathic Arthritis.

**Early school age**											
OligoJIA n = 12					Abn. score	PolyJIA n = 14					Abn. score

	Min	Max	Mean	SD			Min	Max	Mean	SD	

*Disease duration (yrs.)*	.5	5.3	2.7	1.5			.1	5.6	2.3	1.8	
*Disease Outcome:*											
% Swelling index	0	10	1.2	3.1			0	45	12.4	15.4	
% ped. EPM-ROM	0	17	2.6	5			0	60	10.3	15.6	
% CHAQ Ds	0	35	7.9	11.3			0	65	23.2	23.4	
% VAS pain	0	70	13.7	22.8			0	70	17.8	22.1	
% VAS dis. severity	0	75	17.5	23.5			0	70	16.5	23.4	
											
*Motor skills:*						n = 13					
M-ABC (%)	5	93	49	30.8	1		1	96	38.7	33.8	2
											
*Functional skills*											
PEDI Self-care	28.7	54.8	43	8.1	1		18.3	52.1	39.4	8.7	2
PEDI Mobility	10	56.8	28	15.8	8		10	65	30.2	15.8	5
PEDI Social skills	26.6	60.5	41.1	9.1	1		22.6	71.3	44.6	12.9	2

OligoJIA: oligo-articular Juvenile Idiopathic Arthritis, PolyJIA: polyarticular Juvenile Idiopathic Arthritis, ped. EPM-ROM: pediatric Escola Paulista Medicina Range Of Motion Scale, CHAQ Ds: Childhood Health Assessment Questionnaire Disability Score, VAS: Visual Analogue Scale, Dis. Severity: Disease Severity. Disease outcome is presented in % of maximum loss/impairment. M-ABC: Movement Assessment Battery for Children (presented as percentile scores), PEDI: Pediatric Evaluation of Disability Inventory (presented as standard scores, range 30–70). Abn. Score: Abnormal Score (i.e. < -2 SD).

**Table 3:** Disease outcome, level of motor performance and functional skills development in early school aged children with systemic onset- and extended oligoarticular Juvenile Idiopathic Arthritis.

**Early school age**											
systJIA n = 6					Abn. score	Ext-OligoJIA n = 2					Abn. score

	Min	Max	Mean	SD			Min	Max	Mean	SD	

*Disease duration (yrs.)*	.5	2.8	1.4	.9			.5	3.9	2.2	2.4	
*Disease Outcome:*											
% Swelling index	0	15	4.2	5.8			1	20	10.5	13.4	
% ped. EPM-ROM	0	10	4.2	3.7			0	30	15	21.2	
% CHAQ Ds	0	65	20	22.8			10	37	23.5	19.1	
% VAS pain	0	45	13.7	20			5	87	46	57.9	
% VAS dis. severity	0	50	26.5	25.4			0	85	42.5	60.1	
											
*Motor skills:*											
M-ABC (%)	6	93	38.8	31.6			5	79	42	52.3	1
											
Functional skills											
PEDI Self-care	28.6	48	37	6.7	1		35.6	50.7	43.1	10.6	
PEDI Mobility	12.2	48.1	29.5	12	4		10	27.1	18.5	12.1	1
PEDI Social skills	28.7	72	47.2	15.6	1		36.8	39.5	38.1	1.9	

SystJIA: Systemic onset Juvenile Idiopathic Arthritis, Ext-Oligo JIA: extended oligo articular Juvenile Idiopathic Arthritis ped. EPM-ROM: pediatric Escola Paulista Medicina Range Of Motion Scale, CHAQ Ds: Childhood Health Assessment Questionnaire Disability Score, VAS:

Visual Analogue Scale, Dis. Severity: Disease Severity. Disease outcome is presented in % of maximum loss/impairment. M-ABC: Movement Assessment Battery for Children (presented as percentile scores), PEDI: Pediatric Evaluation of Disability Inventory (presented as standard scores, range 30–70). Abn. Score: Abnormal Score (i.e. < -2 SD).

Correlations were calculated between disease course (age at onset, disease duration), disease outcome (joint swelling, joint mobility, physical ability and joint pain), with motor performance and skills development. In PSA children, statistical significant correlations were found between disease duration and pEPM-ROM (Spearman's rho = .49; p < .05); between CHAQ, pEPM-ROM and joint swelling (Spearman's rho = .47; p < .05 and .67; p < .001 respectively), supporting the general notion that disease influences disease outcome. Furthermore, there was a significant correlation between disease outcome on CHAQ and PEDI mobility domain (Spearman's rho = -.59; p < .01), reflecting concurrent constructs of both instruments. The relationship between age at disease onset and BSID_2 _(Spearman's rho = .48; p < .5), is suggestive for the influence of disease on motor performance. In ESA children, comparable with the PSA group, correlations were found between disease duration and disease outcome on pEPM-ROM, joint swelling and CHAQ (Spearman's rho = -.35; p < .05, -.64; p < .0001 and -.45; p < .01, respectively) and age at disease onset, joint swelling and CHAQ Spearman's rho = .50; p < .01, .43; p < .01 respectively). The relationship between disease duration and M-ABC, PEDI social skills domain (Spearman's rho = .46; p < .01 and -.42; p < .01, respectively), and between age at disease onset and PEDI social skills domain (Spearman's rho = .42; p < .01) is suggestive for a disease impact on motor performance and skills and development. Joint swelling and joint pain were significantly correlated with M-ABC (Spearman's rho = -.42; p < .05 and – .37; p < .05). Joint swelling and PEDI social skills scale were correlated (Spearman's rho = -.42; p < .05 and .36; p < .1. These illustrate specific influence of disease outcome parameters on motor performance and skills development. All correlations were regarded to be weak to modest.

## Discussion

This study describes the level of motor performance and the level of acquired functional ability in relation to clinical manifestation and disease outcome in PSA children with JIA and ESA children with JIA. More PSA children showed impairment of motor performance on the BSID_2 _(± 70%) than ESA children on the M-ABC (± 35%). Our finding is in contrast with Hollister's conclusion, thus far the only study to specifically address the topic of motor development in JIA. In Hollister's study no differences were found between the moment 2 children with JIA had reached their motor milestones compared to that of healthy peers [8]. The low number of patients (n = 2) in this case report has most likely biased its conclusion. Our observation that more PSA children compared to ESA children show impaired motor development may well be explained by the fact that motor development at a young age shows higher sequence of consecutive motor skills than at older ages and therefore the impact of the disease on motor performance is much stronger at early ages. Other explanations for this observation could be the fact that motor development is a process of 'jumps and bumps' and that our one-time measurement coincided with a 'bump' [19]. Or that young children tend to adapt much faster to new circumstances than older children do, by that 'self- limiting' their motor behavior, and this may also contribute to the observation that more very young children show impaired motor development. The results from our study however, do stress the need for a longitudinal and systematic inventory of motor development in especially very young children with JIA for at least 5 consecutive years. A greater number of ESA children (55%) compared to PSA children (± 20%) showed impaired functional ability on the PEDI, especially in the mobility domain. This finding is in accordance with the results reported by Miller et al, from a retrospective study where school age children with JIA (with and without manifest synovitis) were compared with healthy controls. His patient population was somewhat older, mean age ± 9.5 years and he reported loss of physical functioning (CHAQ) between 10% and ± 65% measured [7]. In our study the CHAQ and PEDI show sufficient correlation (-.59) to support the conclusion that both groups show comparable functional disability. The CHAQ however is a non- referenced- based, disease specific, outcome measure for childhood rheumatic conditions and in comparison with the PEDI, a reference based generic developmental instrument, is less specific in detecting developmental delay. Our findings therefore allow us to be more precise in the nature and magnitude of the developmental delay in functional abilities. An explanation for the differences found in mobility between PSA and ESA children might be found in the greater demands and expectations for independence in older children together with higher lower extremity involvement which is more common among JIA disease courses than upper extremity involvement [1,20]. Disease course is likely to have an influence on motor performance and functional skills. However, the small number of each subclass of JIA in our two cohorts makes it impossible to draw final conclusions. We also find that duration of the disease or age at onset in both age cohorts were correlated with the outcome on the motor performance levels which leaves room for speculation that disease exposure time may contribute to the motor delay on the BSID_2 _and M-ABC. The complete lack of correlation between motor performance and functional skills need to be further studied. However, speculating on this, one could hypothesize that a child with JIA uses different motor strategies for mastering motor and functional skills. Finally, the distribution of disease subtypes of our patient group was typical for a tertiary centre for pediatric rheumatology, with an overrepresentation of the more severe cases and the more severe subtypes (polyarticular, systemic onset and extended oligo-articular JIA). This could have influenced the outcome on the BSID_2_, M-ABC and PEDI and may have resulted into an overrepresentation of the impairment level, rather than an under-representation in comparison with the JIA population in general. However, the over-representation of girls in this study population is in agreement with the literature on gender distribution in JIA and was therefore of no concern to the researchers [1,20].

## Conclusion

In a study of 56 children with JIA, the PSA children with JIA suffered more from impaired motor performance than from impaired functional skills, while ESA children with JIA showed more impairment in the performance of functional skills. Disease onset and disease duration show a correlation with motor performance in both cohorts. Motor performance does not seem to be correlated with performance of functional skills in both PSA- and ESA children with JIA. The results from this study draw the attention to an area not yet frequently studied, i.e. motor performance and functional skills development in childhood chronic arthritis. Clinicians involved in the follow up of these patients should be aware of the functional impact joint disease has as well as the feasibility to reliable measure this outcome. Those who are responsible for the rehabilitation of PSA- and ESA children should be aware of the different impact joint disease has in these age groups and design therapeutic programs accordingly. Future research should use longitudinal designs and focus on predictors of motor delay and delay in development of functional skills to further improve the efficacy of rehabilitation programs and follow up protocols.
